# Theoretical Study for High-Energy-Density Compounds Derived from Cyclophosphazene. IV. DFT Studies on 1,1-Diamino-3,3,5,5,7,7-hexaazidocyclotetraphosphazene and Its Isomers

**DOI:** 10.3390/ijms10083502

**Published:** 2009-08-06

**Authors:** Jianguo Zhang, Huihui Zheng, Tonglai Zhang, Man Wu

**Affiliations:** State Key Laboratory of Explosion Science and Technology, Beijing Institute of Technology, Beijing 100081, China

**Keywords:** density functional theory, vibrational analysis, Mulliken analysis, heats of formation, diamino-hexaazidocyclotetraphophazene

## Abstract

In the present study, a theoretical study of 1,1-diaminohexaazidocyclotetraphophazene (DAHA) and its isomers has been performed, using quantum computational density functional theory (B3LYP and B3PW91 methods) with 6-31G* and 6-31G** basis sets implemented in Gaussian 03 program suite. Molecular structure and bonding, vibrational frequencies, Milliken population analysis, and natural bond orbit (NBO) have been studied. The heats of formation from atomization energies have also been calculated based on the optimized geometry. The obtained heats of formation data are compared with their homologous cyclophosphazene in order to demonstrate the accuracy of the methods, which indicate that the studied compounds might be potentially used as high energetic materials. In addition, the relative stability of five isomers have been deduced based on the total energy and the gap of frontier orbital energies.

## 1. Introduction

Research involving the search for and synthesis of new high energy (HE) compounds is an ongoing quest. There are many molecules that fall into this category, and analysis of their characteristics yields information about trends in structure and chemistry. These trends include strained ring structures, molecules that are unstable with respect to their combustion products, and the inclusion of various phosphazene ring containing functional groups, such as azido, nitro and amino groups. Based on these traits, a phosphazene ring containing amino and azido groups would be a kind of possible high-energy compound.

Compounds that contain azido groups are most often highly energetic and some tend to be unstable. However, over the last years, relatively stable azido substituted carbon-nitrogen heterocycles with densities >1.7 g·cm^−3^ and with very high heats of formation have been reported [1]. Until now, most research has been based on compounds with both carbon-azido and carbon-amino or nitrogen-amino substituents [2,3], and the structure and properties of energetic materials with phosphorus-azido and phosphorus-amino moieties have been only rarely discussed in the scientific literature [4].

Cyclophosphazene derivatives have attracted much interest and have been reviewed over the years [5,6], not only from the synthetic and mechanistic points of view but also with respect to their unusual structural characteristics and their place on the borderline between inorganic and organic high polymer chemistry. Numerous reactions of chlorocyclophosphazenes with amino groups have been investigated [5–7]. Chaplin *et al.* [8] revisited the electronic structures of a series of phosphazene compounds and reported the natural bond orbital and topological electron density analyses. In addition, they investigated the substituent effects on the P-N bond and the aromaticity of these N-P compounds. Diaminohexaazidocyclotetraphophazene [N_4_P_4_(N_3_)_6_(NH_2_)_2_] is such a material recently synthesized as a primary explosive for use in munitions. This novel compound has the impact, friction, and electrostatic properties of a strong primary explosive. 1,1-Diamino-3,3,5,5,7,7-hexaazidocyclotetraphosphazene and *trans*-1,5-diamino-1,3,3,5,7,7-hexaazidocyclotetraphosphazene which have been shown to be useful as an energetic composition and a percussion primer have been synthesized and reported [9]. However, the related synthesis and characterization of the other isomers, *e.g.,* 1,3-diamino-1,3,5,5,7,7-hexaazidocyclotetraphosphazene (*cis*- and *trans*-) and *cis*-1,5-diamino-1,3,3,5,7,7-hexaazidocyclotetraphosphazene haven’t been reported, to the best of our knowledge.

**Figure 1.**
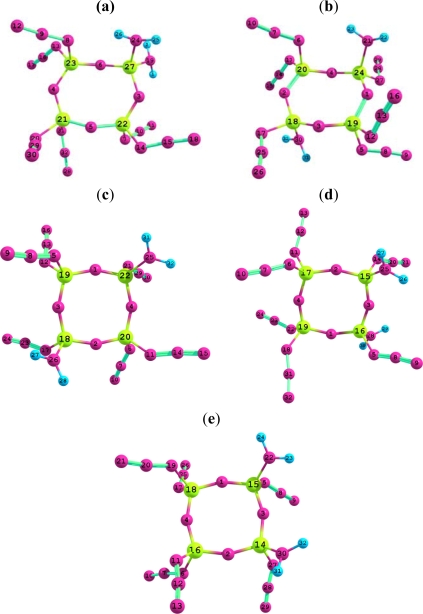
The structure and the atom serial number of five isomers for diamino-hexaazidocyclo- tetraphosphazene (the yellow, pink and blue balls denote phosphorus nitrogen, and hydrogen atoms, respectively). (a) 1,1-Diamino-3,3,5,5,7,7-hexaazidocyclotetraphosphazene (**I**); (b) *trans*-1,5-diamino-1,3,3,5,7,7-hexaazidocyclotetraphosphazene (**II**); (c) *cis*-1,5-diamino-1,3,3,5,7,7-hexaazidocyclotetraphosphazene (**III**); (d) *trans*-1,3-diamino-1,3,5,5,7,7-hexaazidocyclotetraphosphazene (**IV**); (e) *cis*-1,3-diamino-1,3,5,5,7,7-hexa-azidocyclotetraphosphazene (**V**).

In previous publications, we have ever reported the preparation, structural characterization, and theoretical studies of 1,1-spiro(ethylenediamino)-3,3,5,5-tetrachlorocyclotriphosphazene and its nitration product [10]. In addition, the high nitrogen-contented energetic compounds 1,1,3,3,5,5,7,7-octaazidocyclotetraphosphazene [11] and 1,1,3,3,5,5-hexaazidocyclotriphosphazene [12] have been investigated using the quantum chemistry method. As a part of a series of research works on high-energy-density compounds derived from cyclophosphazene, we have performed theoretical studies on the five isomers diaminohexaazidocyclotetraphosphazene (*i.e.,* 1,1-diamino-3,3,5,5,7,7-hexa-azido-cyclotetraphosphazene (**I**), *trans*-1,5-diamino-1,3,3,5,7,7-hexaazidocyclotetraphosphazene (**II**), *cis*-1,5-diamino-1,3,3,5,7,7-hexaazidocyclotetraphosphazene (**III**), *trans*-1,3-diamino-1,3,5,5,7,7-hexa-azidocyclotetraphosphazene (**IV**) and *cis*-1,3-diamino-1,3,5,5,7,7-hexaazidocyclotetraphosphazene (**V**), Figure 1). We have mainly studied the structures and bonding of the isomers to investigate their thermodynamic stabilities and bonding natures. In this study, density functional theory (DFT) B3LYP and B3PW91 methods have been used with a variety of basis sets using Gaussian 03 program suite [13].

## 2. Computational Methods

All of the structures were fully optimized without any symmetry limitation using the B3LYP [14] and B3PW91 methods with 6-31G* and 6-31G** basis sets. A comparative study on the effect of basis set on the calculated structures has also been carried out. To characterize the nature of the stationary points and to determine the zero-point vibrational energy corrections, harmonic vibrational analyses were performed subsequently on each optimized structure at the same level with the same basis set. Further, the natural bond orbit (NBO) analysis [15] was calculated at the B3LYP/6-31G** level to help us understand the interactions between the different orbits. Finally, the heats of formation of the title compounds were calculated using standard statistical thermodynamic methods. To compare the relative stability of the five isomers, we calculated their total energy and frontier orbital energy.

## 3. Results and Discussion

### 3.1. Structural analysis

For there is no obvious deviation in optimized parameters for the five isomers, Table 1 gives part of the optimized parameters of 1,1-diamino-3,3,5,5,7,7-hexaazidocyclotetraphosphazene as an example.

We can see from the Table 1 that the two DFT methods used in this study give similar bond length and bond angle values. Use of a polarized basis set has a negligible effect on the structural parameters. As shown in the Table, in the eight-numbered phosphorus-nitrogen ring, the P-N bond length is between 1.580 −1.621 Å, with an average length of 1.596 Å, which lies between the typical single and double bonds bond lengths. There is no obvious alternating single and double bond in the ring, in agreement with the identical P-N bond found in (NPX_2_)_4_ (X=H or halogens) [16]. In addition, the distances of all P-N bonds aren’t the same, which is caused by the interaction of the nitrogen lone pair of the ligands with the P-N bonds [17]. The P-N bond conjoint to −N_3_ is longer than that conjoint to −NH_2_, with the maximum size of 0.057 Å; this can be attributed to the different electro-negativity of different substituents on the phosphorus atom. In addition, we can see that the N-H bond is the smallest and the P-N_α_ bond is larger than any of the others, suggesting that the P-N_α_ bonds are the weakest and will easily break under the outside force. The bond angles about the P and N centers are also well described. The P-N-P angles angle of 135° are larger than the N-P-N angle of 125°, while the N-P-N of 115° adjacent to –NH_2_ is smaller than the other N-P-N angles. The P-N-P angles range from 133° to 141° and the N-P-N are approximately 120°, regardless of the different substituent and the location of the amino group, which indicate that the P-N-P angle is more flexible, and this is in agreement with the literature [6,18]. In addition, we know that the structure of the phosphazene ring displays a contorted chair form from the torsion angles for N and P atoms in the ring in Table 1.

### 3.2. Vibrational analysis

As examples Table 2 presents the calculated vibrational frequencies from gas phase IR spectra of the 1,1-diamino- and *trans*-1,5-diamino-compounds without any scale factor. The absence of any imaginary frequency indicates that all of the optimized structures correspond to the minimum point on the intra-molecular potential energy surface.

Analysis of the calculated vibrational frequencies shows that the frequencies of the five compounds show no large deviations. We find that the molecules have a sharp peak between 3,500 cm^−1^−3,700 cm^−1^ correspond to the stretching vibration of the N-H bond. The modes rang from 22,00 cm^−1^−2,350 cm^−1^ and 1,300 cm^−1^−1,360 cm^−1^ have been identified as the stretching vibrations of the −N_3_ group, while the peaks at 1,266 cm^−1^ (1,689 cm^−1^) and 1,371 cm^−1^ (748 cm^−1^) are attributed to the in-plane stretching of P-N-P bonds, which are in agreement with the P-N-P stretch seen in (NPF_2_)_4_ [19]. The low vibration (470 cm^−1^−920 cm^−1^) can be assigned to the bend of −NH_2_ group and the torsion of −N_3_ group. Above the analysis, we can found that the strong absorption peak is between 1,250 cm^−1^ −1,350 cm^−1^, which is assigned to the phosphorus-nitrogen ring, and the other strong peak around 2300 cm^−1^, which is assigned to the N_β_-N_γ_ unsymmetrical stretching. It is clearly seen that these groups have corresponding IR signatures, which. are expected to provide useful information for their further experimental detections.

### 3.3. NBO analysis

Tables 3–5 give the NBO analysis of the five isomers based on B3LYP/6-31G**. Here, in the NBO analysis [20], the donor-acceptor interactions are estimated by second-order perturbation theory. The stabilization energy E(2) for each donor NBO(*i*) and acceptor NBO (*j*) are associated with *i* → *j* delocalization, which is estimated by the following equation:

$$E\left(2\right)=\frac{q_{i}F{\left(i,j\right)}^{2}}{{ɛ}_{j}-{ɛ}_{i}}$$

From the data, we can conclude that the position of the amino group has an effect on the stabilization energy. By analysing the data, we find the strongest interaction mainly occurs on the lone pair of N_α_ and the π anti-bond orbit of N_β_-N_γ_. There are weak mutual interactions among the P-N bonds in the phosphazene ring, which indicate that the P-N bonds have a tendency to offer electrons to each other and there are weak conjugated interactions in the ring. Between the σ orbit of P-N_α_ bonds and the lone pair of N atom in the ring there also exist weak interactions; we consider there is conjugated interaction. In the 1,5-diaminohexaazidocyclotetraphosphazene molecule it can be found that there are stronger interactions existing between the P-N in the eight-member ring and the P-N_α_ bond, the two P-N_α_ bonds linked on the same phosphorus atom also have strong mutual interaction and the maximal interaction between σ*_P-Nα_ and π*_P-Nα_. It indicates that there exists a strong repulsion between two azido groups. In addition, the interaction between the P-N_α_ bond and the N_β_-N_γ_ bond is also strong, but the interaction between P-N (amino) and P-N_α_ is weaker than that between two P-N_α_ bonds, it also indicate that amino group and azido group linked on the same phosphorus atom is more stable than that two azido groups link on one phosphorus, so we can infer that the P-N_α_ bond is unstable and easy to separate from the ring. In the 1,3-diaminohexaazidocyclotetraphosphazene molecule, the interaction mainly occur between σ*_P-N_ and σ*_P-N_ in the ring, the n_Nα_ → π*_Nβ-Nγ_ interaction between the lone pair of the N_α_ and π*_Nβ-Nγ_ anti-bond is also stronger than other interactions. There exist a N≡N at the end of the azido groups. The interactions between the σ_P-Nα_ bond and the σ*_P-Nα_ anti-bond, σ_P-N_ (amino) bond and σ*_P-Nα_ anti-bond are all about 3.20 kcal·mol^−1^, weaker than that in 1,5-diaminohexaazidocyclo-tetraphosphazene. It also indicates the two azido groups are more stable than in 1,5-diamino-hexaazidocyclotetraphosphazene. There is weak interaction between the P-N_α_ bond and the separated P-N (ring) bonds, which show there is conjugated interaction in the phosphazene ring and the whole structure is stable. From the stabilization energies between these bonds, we can conclude that the interaction in the 1,1-diaminohexaazidocyclotetraphosphazene molecule is stronger and there exists a strong repulsion between two azido groups linked on the same phosphorus atom and the molecule is unstable.

From the data, we can conclude that the position of the amino group has an effect on the stabilization energy. By analysing the data, we can find the strongest interaction mainly occur on the lone pair of N_α_ and the π anti-bond orbit of N_β_-N_γ_. There are weak mutual interactions among the P-N bonds in the phosphazene ring, which indicate that the P-N bonds have the tendency to offer electrons to each other and there is weak conjugated interactions in the ring. Between the σ orbit of P-N_α_ bonds and the lone pair of N atom in the ring there also exist weak interactions, so we consider there is a conjugated interaction. In the 1,5-diaminohexaazidocyclotetraphosphazene molecule, it can be found there are stronger interactions existing between the P-N in the eight-member ring and the P-N_α_ bond, the two P-N_α_ bonds linked on the same phosphorus atom also have a strong mutual interaction and the maximal interaction is between σ*_P-Nα_ and π*_P-Nα_. It indicates that there exists a strong repulsion between the two azido groups. In addition, the interaction between the P-N_α_ bond and the N_β_-N_γ_ bond is also strong, but the interaction between P-N (amino) and P-N_α_ is weaker than that between the two P-N_α_ bonds; this also indicates that amino group and azido group linked on the same phosphorus atom are more stable than that two azido groups linked on one phosphorus, so we can infer that the P-N_α_ bond is unstable and easy to break from the ring. In the 1,3-diaminohexaazidocyclo-tetraphosphazene molecule the interaction mainly occurs between σ*_P-N_ and σ*_P-N_ in the ring and the n_Nα_→π*_Nβ-Nγ_ interaction between the lone pair of the N_α_ and π*_Nβ-Nγ_ anti-bond is also stronger than other interactions. There exists an N≡N at the end of the azido groups. The interactions between σ_P-Nα_ bond and σ*_P-Nα_ anti-bond, σ_P-N_ (amino) bond and σ*_P-Nα_ anti-bond are all about 3.20 kcal·mol^−1^, weaker than that in 1,5-diaminohexaazidocyclotetraphosphazene. It also indicates the two azido groups are more stable than that in 1,5-diaminohexaazidocyclotetraphosphazene. There is a weak interaction between the P-N_α_ bond and the separated P-N (ring) bonds, which shows there is a conjugated interaction in the phosphazene ring and the whole structure is stable. From the stabilization energies between these bonds, we can conclude that the interaction in the 1,1-diaminohexaazidocyclotetraphosphazene molecule is stronger and there exists strong repulsion between two azido groups linked on the same phosphorus atom and the molecule is unstable.

### 3.4. Mulliken overlaps population analysis and the atomic charges

Bond overlap populations can reflect the electron accumulations in the bonding region, and they can provide us detailed information about the chemical bonding. As a whole, the larger the population is, the greater the bond overlaps. Though the Mulliken population analysis suffers from some shortcomings, however, for the purpose of comparing trends in the electron distribution for homologous compounds at the same calculation condition, results derived from Mulliken population analysis are still meaningful. Table 6 gives the ranges of the overlap populations for the five isomers of Diaminohexaazidocyclotetraphophazene based on B3LYP/6-31G**.

Inspecting the overlap populations in Table 6, it can be found that the variations of bond overlap populations are consistent with those of the geometric parameters, and they are also affected by the relative positions of amino groups. When two amino groups substitute on the same phosphorus atom, from the population of P-N bonds in the ring, we can find that the P-N bonds (ring) adjacent to amino groups have smaller populations than that adjacent to azido groups, while populations of P-NH_2_ bonds are the strongest compared with other isomers. This can be attributed to the intra-molecular interactions between amino groups and ring. When two amino groups substitute on a different phosphorus atom, populations of P-N_α_ in *trans*-structures are larger than that in *cis*-structures. Besides, we can see that the P-N_α_ bonds have the smallest populations and the N_β_-N_γ_ bonds have the largest. Although the Mulliken populations fail to give reliable characterization of bond strength, they obviously demonstrate that the P-N_α_ bonds are the weakest and the N_β_-N_γ_ bonds are the strongest, we can infer that the P-N_α_ bonds are more fragile than other bonds in the molecules and the P-N_α_ bonds could be ruptured initially in their thermal decompositions. Comparing the populations of P-N (amino) with P-N_α_ (azido), we can find the latter have smaller populations than the former, which demonstrate the bonds joined to amino groups are more stable than those joined to azido groups. This may explained why the azido compounds are highly energetic and can be ruptured by outside stimuli. Finally, from the populations, we can conclude the relative stability of the bonds in the molecules is: P-N_α_ < N_α_-N_β_ < P-NH_2_ < P-N(ring) < N_β_-N_γ_.

The ranges and the average charge of the same kind of atoms for the five isomers of diaminohexaazidocyclotetraphophazene based on B3LYP/6-31G** are summarized in Table 7. It can be found that phosphorus atomic charges (1.057–1.089 at DFT B3LYP/6-31G** level of theory) are less positive, whereas the nitrogen in the cyclotetraphosphazene (−0.683 to −0.602 at the same lever of theory) and α-nitrogen (−0.537 to −0.449) ones are less negative, so we can conclude that some charges are transferred from phosphorus atoms to nitrogen atoms in the cyclotetraphosphazene [8,18]. In the azido group, the β-nitrogen display some positive atomic charges (0.435–0.480), however the α- and γ-nitrogen have some negative atomic charges. In addition, the α-nitrogen has more negative atomic charges than the γ-nitrogen because of the electronic effect from the phosphorus.

### 3.5. Heats of formation from computed atomization energies

The calculation of theoretical heats of formation (HOFs) is essential for many of the applications of quantum chemistry, in particular for aiding in the interpretation of experimental results and for the prediction of reaction kinetics. Several theoretical procedures such as group additive method; molecular mechanics and semi-empirical MO methods have been used to estimate heats of formation. In this study, DFT methods and basis sets are selected to calculate the heats of formation through the selected atomization energies. Experimental heats of formation of element P, N, H and standard temperature corrections can be found in the literature [21,22].

The HOFs of the five isomers in the gas phase at 298.15K are predicted using B3LYP and B3PW91 methods with 6-31G**. The results are presented in Table 8; also included are the calculated HOFs and experimental data [23] of N_3_P_3_(N_3_)_6_. The two levels of theory show the same trend for the relative stabilities of the title compounds, the calculated HOFs are all positive, which indicate that the compounds are unstable in the gas phase. For there are no corresponding experimental data of the title compounds, we can only make comparison with the calculated and experimental data HOFs of their homologous cyclophosphazene N_3_P_3_(N_3_)_6_. By comparing the data, it is noted that the HOFs calculated by B3LYP and B3PW91 are both close to the experimental results, the deviation among the five isomers is no more than 3 kcal mol^−1^, which demonstrates that the method we used is accurate, and the HOFs of studied compounds are not sensitive to the position of amino groups. From above analysis, we conclude that the isomers for diaminohexaazidocyclotetraphosphazene have high heats of formation and high nitrogen-contents, so they tend to be a kind of potential high-energetic-material, with the accompanying tendency to be sensitive to friction, impact and heat.

### 3.6. Relative total energies and the frontier orbital energies for the compounds **I**–**V**

Because of the different positions of the amino groups, there exist five isomers of diaminohexaazidocyclotetraphosphazene. To estimate the relative stability of compounds **I**–**V**, we summarize their related total energy data and the frontier orbital energies in Table 9. From the energy data, we can find that the total energy of *cis*-1,3-diaminohexaazidocyclotetraphosphazene (**II**) is lowest and the Δ E_L-H_ is biggest. On the contrary, the total energy of 1,1-diamino-3,3,5,5,7,7-hexaazidocyclo-tetraphosphazene (**I**) is the highest and the ΔE_L-H_ is least among those of all isomers. So, we can conclude that *cis*-1,3-diaminohexaazidocyclotetraphosphazene (**II**) is most stable and 1,1-diaminohexaazidocyclotetraphosphazene is the most unstable among all isomers, and their expected sequence of stability is **II** > **III** > **IV** > **V** > **I**, which is consistent with the experimental results [9,10]. The initial decomposition temperature of the isomer **IV** is higher than that of the isomer **I**.

## 4. Conclusions

Density functional theory B3LYP and B3PW91 calculations have been carried out on the five isomers of diaminohexaazidocyclotetraphosphazene. The structural investigation results show that the title molecules do not have planar structures. There are no alternating single and double bonds in the phosphazene ring and the P-N bond lengths are not identical, which indicate the molecules have no aromaticity. The different positions of the amine group have no large effect on the optimized parameters. The Mulliken population analysis indicates that the P-N_α_ bonds are the weakest and could be ruptured initially by stimuli, the relative stability of the bonds in the molecules is: P-N_α_ < N_α_-N_β_ < P-NH_2_ < P-N(ring) < N_β_-N_γ_. Besides, different substituents affect the stability of the P-N bonds in the ring. For thermo-chemical analysis on the optimized structures, it can be predicted that they all have high heats of formation and unstable in the gas phase, so they might be potential energetic materials. In addition, we concluded the relative stability of five isomers according to their total energy and the gap of frontier orbital energies.

## Figures and Tables

**Table 1.** The selected bond lengths (Ǻ), bond angles (°) and dihedral angle (°) of 1,1-diamino-3,3,5,5,7,7-hexaazidocyclotetraphosphazene (**I**) #.

**Structure parameter**	B3LYP/6-31G*	B3LYP/6-31G**	B3PW91/6-31G*	B3PW91/6-31G**
Bond lengths				
N4-P23	1.599	1.599	1.596	1.596
N3-P27	1.621	1.621	1.619	1.619
N3-P22	1.580	1.579	1.577	1.578
N6-P27	1.609	1.610	1.606	1.605
N5-P22	1.583	1.583	1.580	1.581
P21-N4	1.592	1.592	1.589	1.589
P21-N_α_	1.709	1.723	1.716	1.716
N_α_-N_β_	1.240	1.240	1.235	1.235
P27-NH_2_	1.667	1.666	1.663	1.661
N_β_-N_γ_	1.135	1.135	1.133	1.133
N19-H1	1.015	1.013	1.014	1.012
Bond angles				
P21-N4-P23	135.9	135.9	135.4	135.4
N4-P21-N5	120.7	120.6	120.5	120.5
N_α_-P21-N_α_	101.4	101.4	101.5	101.5
P21-N_α_-N_β_	118.3	118.3	118.3	118.3
N_α_-N_β_-N_γ_	174.2	173.7	174.2	174.2
H1-N19-H2	113.2	113.6	113.4	113.9
N3-P22-N5-P21	68.9	68.8	68.5	68.4
P22-N5-P21-N4	−70.1	−70.0	−70.3	−70.3
N5-P21-N4-P23	26.1	26.1	26.3	26.4
P21-N4-P23-N6	20.8	20.7	20.3	20.5
N4-P23-N6-P27	−87.1	−87.2	−88.0	−87.9
P23-N6-P27-N3	75.9	75.7	75.3	75.7

# In the table, N_α_ is the nitrogen atom in the azido group which is connected directly with the P atom, and the N_β_ is the middle nitrogen atom in the azido group, the top nitrogen atom is N_γ_.

**Table 2.** Some main vibrational harmonic frequencies in cm^−1^ and their IR intensities in km mol^−1^ (given in parentheses), calculated for the optimized structures of the isomers of diaminohexaazidocyclotetraphosphazene using the B3LYP/6-31G** level of theory.

**ν**	**I**	**II**	**III**	**IV**	**V**	**Assignment**
1	390.2 (96)	406.4 (57)	478.4(129)			P-N ring in-plane twist
2	540.9 (184) 550.2 (211)	542.2 (215) 552.4 (76)				P-N ring twist N-H bending, –N_3_ twist
3	563.6 (294)	576.1 (106)		563.1(214)	556.5(188)	−N_3_ torsion, N-H bending
4	578.8 (22) 612.5 (234)	590.9 (238) 608.8 (230)			610.8(418)	−N_3_ twist, torsion
5	689.6 (266)	754.4 (210)	709.4(275)	758.7(397)		N_α_-N_β_ in plane twist
6	802.2 (176)	821.1 (196)				N-H unsymmetrical twist
7	939.6 (126)	923.4 (217)	920.3(149)	913.7(170)	915.9(129)	P-NH_2_ in-plane stretching
8	1,034.3 (68) 1,049.9 (75)	1,020.1 (25) 1,014.2 (53)			941.7(148)	N-H in-plane unsymmetrical wag
9	1,266.3 (1689)	1,281.8 (1775)	1,270.4(1847)	1,293.2(1980)	1,296.6(2017)	P-N-P in-plane stretching
10	1,313.8 (298) 1,318.7 (380) 1,326.1 (337) 1,341.8 (104)	1,314.8 (134) 1,321.0 (461) 1,336.8 (431) 1,349.2 (128)	1,338.3(318)	1,315.5(1282) 1,322.9(479)	1,319.0(380)	N_α_-N_γ_ symmetrical stretching
11	1,330.3 (1155)	1,307.8 (1553)	1,311.0(1096)	1,326.7(389)	1,299.3(2080)	P-N-P in-plane stretching
12	1,371.5 (748)	1,364.5 (586)	1,367.2(632)	1,380.8(915)		P-N-P symmetrical stretching
13	1,592.4 (100)	1,587.5 (95)				−NH_2_ in-plane bending
14	2,277.9 (533) 2,299.4 (607) 2,309.1 (1046)	2,295.1 (213) 2,295.4 (1004) 2,300.0 (1078)	2,292.2(359) 2,295.8(588) 2,308.1(858)	2,286.9(781) 2,296.4(1006) 2,300.1(730)	2,286.7(730) 2,294.7(496) 2,300.8(631)	N_β_-N_γ_ unsymmetrical stretching
15	3,542.0 (52) 3,566.2 (37)	3,539.0 (47) 3,562.6 (41)	3,562.9(66)	3,530.4(61)	3,574.3(56)	N-H symmetrical stretching
16	3,660.2 (66) 3,679.4 (39)	3,651.2 (57) 3,680.7 (36)	3,681.1(35)	3,683.9(38)	3,694.5(40)	N-H unsymmetrical stretching

**Table 3.** Part of calculated results of 1,1-diamino-3,3,5,5,7,7-hexaazidocyclotetraphosphazene (**I**) by NBO analysis.

**Donor NBO (i)**	**Acceptor NBO (j)**	**E(2)/(kcal·mol^−1^)**
LP(2)N7	BD*(2)N10-N11	113.83
LP(2)N7	BD*(1)N5-P22	2.30
LP(2)N7	BD*(1)N3-P22	0.87
LP(1)N7	BD*(3)N10-N11	5.77
LP(2)N8	BD*(2)N9-N12	106.13
LP(2)N8	BD*(1)N4-P23	5.65
LP(2)N8	BD*(1)N6-P23	2.50
LP(2)N13	BD*(2)N16-N17	106.72
LP(2)N14	BD*(2)N15-N18	107.55
LP(2)N20	BD*(2)N29-N30	107.18
LP(2)N31	BD*(2)N28-N32	104.22
BD(1)N20-P21	BD*(1)N5-P22	2.63
BD(1)N20-P21	BD*(1)N4-P23	1.77
BD(1)N19-P27	BD*(1)N6-P23	1.76
BD(1)N19-P27	BD*(1)N3-P22	1.49
BD(1)N3-P22	BD*(1)N5-P21	0.88
BD(1)N4-P21	BD*(1)N5-P21	1.01
BD(1)N7-P22	BD*(3)N10-N11	17.97
BD(1)N8-P23	BD*(3)N9-N12	16.35
LP(2)N3	BD*(1)N7-P22	23.17
LP(2)N3	BD*(1)N24-P27	12.98
LP(2)N4	BD*(1)N8-P23	19.13
LP(2)N4	BD*(1)P21-N31	15.62
LP(1)N19	BD*(1)N6-P27	12.95
LP(1)N24	BD*(1)N3-P27	12.40
BD*(1)N4-P23	BD*(1)N6-P23	24.07
BD*(1)N6-P27	BD*(1)N6-P23	53.73
BD*(1)N3-P27	BD*(1)N3-P22	46.89

**Table 4.** NBO analysis results of 1,5-diamino-1,3,3,5,7,7-hexaazidocyclotetraphosphazene (**II** and **III**).

**Compounds**	**Donor NBO (i)**	**Acceptor NBO (j)**	**E(2)/(kcal·mol^−1^)**
*trans-1,5-diamino-1,3,3,5,7,7-hexaazidocyclotetraphosphazene***(II)**			
	BD(1)N1-P19	BD*(2)N5-P19	47.76
	BD(1)N1-P19	BD*(1)N12-P19	30.31
	BD(1)N3-P19	BD*(2)N12-P19	82.96
	BD(1)N3-P19	BD*(1)N5-P19	20.46
	BD(1)N2-P20	BD*(2)N6-N20	50.05
	BD(1)N2-P20	BD*(1)N11-P20	32.63
	BD(1)N2-P18	BD*(1)N17-P18	2.08
	BD(1)N1-P22	BD*(1)N21-P22	4.97
	BD(1)N4-P22	BD*(1)N21-P22	1.44
	BD(1)N4-P22	BD*(2)N21-P22	94.77
	BD*(1)N5-P19	BD*(2)N12-P19	109.85
	BD*(1)N25-P22	BD*(2)N21-P22	34.99
	LP(2)N17	BD*(2)N23-N24	111.66
*cis-1,5-diamino-1,3,3,5,7,7-hexaazidocycl-tetraphosphazene***(III)**			
	BD(1)N2-P20	BD*(2)N6-N20	49.58
	BD(1)N2-P20	BD*(1)N11-P20	32.61
	BD(1)N4-P20	BD*(2)N11-P20	80.24
	BD(1)N3-P19	BD*(2)N12-P19	84.57
	BD(1)N1-P19	BD*(2)N5-P19	50.43
	BD(1)P18-N3	BD*(2)N17-P18	11.84
	BD(1)P18-N3	BD*(1)N30-P18	11.37
	BD(1)N1-P24	BD*(1)N21-P24	3.80
	BD(1)N4-P24	BD*(2)N27-P24	55.88
	BD(2)N5-P19	BD*(2)N8-N9	95.82
	BD*(2)N17-P18	BD*(1)N30-P18	72.58
	BD*(2)P24-N27	BD*(1)N21-P24	116.83

**Table 5.** NBO analysis results of 1,3-diamino-1,3,5,5,7,7-hexaazidocyclotetraphosphazene (**IV** and **V**).

**Compounds**	**Donor NBO (i)**	**Acceptor NBO (j)**	**E(2)/(kcal·mol^−1^)**
*trans-1,3-diamino-1,3,5,5,7,7-hexaazidocyclotetraphosphazene* (**IV**)			
	LP(2)N5	BD*(2)N8-N9	110.94
	LP(2)N18	BD*(2)N31-N32	108.18
	LP(2)N6	BD*(2)N7-N10	105.75
	BD*(1)N1-P16	BD*(1)N1-P19	102.54
	BD*(1)N2-P15	BD*(1)N2-P17	97.4
	BD*(1)N5-P16	BD*(1)P16-N28	2.28
	BD*(1)N14-P15	BD*(1)P15-N25	3.95
	BD*(1)P16-N28	BD*(1)N3-P15	3.28
	BD*(1)N18-P19	BD*(1)P19-N22	4.11
	BD*(1)P19-N22	BD*(1)N1-P16	2.08
	BD*(1)P19-N22	BD*(1)N4-P17	3.27
	BD*(3)N23-N24	BD*(1)N23-N24	9.52
	BD*(3)N23-N24	BD*(1)P19-N22	6.03
*cis-1,3-diamino-1,3,5,5,7,7-hexaazidocyclotetraphosphazene* (**V**)			
	BD(1)N17-P18	BD*(1)N19-P18	3.20
	BD(1)P14-N27	BD*(1)N30-P14	3.21
	BD(1)N5-P15	BD*(1)N22-P15	3.22
	BD(1)N5-P15	BD*(1)N5-P19	20.46
	BD(1)N5-P15	BD*(3)N8-N9	16.04
	LP(2)N19	BD*(2)N20-N21	105.23
	LP(1)N22	BD*(1)N5-P15	12.77
	LP(2)N27	BD*(2)N28-N29	112.74
	LP(2)N27	BD*(1)N30-P14	10.01
	LP(1)N30	BD*(1)N27-P14	14.36
	BD*(1)N1-P15	BD*(1)N1-P18	99.55
	BD*(1)N2-P14	BD*(1)N2-P16	90.33
	BD*(3)N25-N26	BD*(1)N17-P18	7.12
	BD*(3)N28-N29	BD*(1)N28-N29	10.3

**Table 6.** Ranges of the Bond Overlap Population for five isomers of diaminohexaazido-cyclotetraphosphazene at the B3LYP/6-31G** level of theory.

**Isomers**	**P-N(ring)**	**P-N_α_**	**N_α_-N_β_**	**N_β_-N_γ_**	**P-N(amino)**	**N-H**
**I**	0.383 ~ 0.510	0.201 ~ 0.275	0.265 ~ 0.301	0.586 ~ 0.601	0.350 ~ 0.353	0.341 ~ 0.345
**II**	0.442 ~ 0.501	0.210 ~ 0.271	0.264 ~ 0.294	0.597 ~ 0.601	0.306 ~ 0.352	0.341 ~ 0.344
**III**	0.445 ~ 0.498	0.219 ~ 0.276	0.266 ~ 0.303	0.595 ~ 0.597	0.310 ~ 0.353	0.341 ~ 0.347
**IV**	0.454 ~ 0.495	0.217 ~ 0.273	0.272 ~ 0.313	0.594 ~ 0.600	0.295 ~ 0.343	0.338 ~ 0.344
**V**	0.438 ~ 0.493	0.207 ~ 0.268	0.264 ~ 0.327	0.596 ~ 0.599	0.320 ~ 0.342	0.343 ~ 0.346

**Table 7.** The atomic charges from the Mulliken population analysis for five isomers of diaminohexaazidocyclo-tetraphosphazene at the B3LYP/6-31G** level of theory ##.

**Isomers**	**I**	**II**	**III**	**IV**	**V**
**P (ring)**	1.070 ~ 1.089 1.078	1.062 ~ 1.072 1.068	1.061 ~ 1.071 1.066	1.057 ~ 1.068 1.063	1.062 ~ 1.070 1.067
**N (ring)**	−0.683 ~ −0.602 −0.640	−0.639 ~ −0.630 −0.635	−0.649 ~ −0.622 −0.635	−0.683 ~ −0.619 −0.636	−0.645 ~ −0.621 −0.633
**N_α_**	−0.537 ~ −0.472 −0.491	−0.530 ~ −0.449 −0.476	−0.475 ~ −0.471 −0.473	−0.472 ~ −0.470 −0.471	−0.472 ~ −0.469 −0.478
**N_β_**	0.458 ~ 0.480 0.465	0.437 ~ 0.460 0.444	0.438 ~ 0.452 0.447	0.435 ~ 0.453 0.447	0.437 ~ 0.453 0.448
**N_γ_**	−0.257 ~ −0.216 −0.228	−0.233 ~ −0.211 −0.224	−0.234 ~ −0.214 −0.228	−0.251 ~ −0.213 −0.226	−0.238 ~ −0.212 −0.223
**N (amino)**	−0.861 ~ −0.818 −0.840	−0.829 ~ −0.813 −0.821	−0.821 ~ −0.821 −0.821	−0.823 ~ −0.823 −0.823	−0.823 ~ −0.820 −0.822
**H (amino)**	0.348 ~ 0.379 0.361	0.355 ~ 0.375 0.362	0.359 ~ 0.362 0.361	0.359 ~ 0.360 0.360	0.355 ~ 0.361 0.357

## In the table, the first line number is the charge range of the same kind of atoms and the second line number is their average value in every cell.

**Table 8.** Calculated HOFs for five isomers of diaminohexaazidocyclotetraphosphazene (kcal•mol^−1^) from atomization energy at 298.15 K.

**Compounds**	B3LYP/6-31G**	B3PW91/6-31G**	**Expt [24]**
**I**	424.03	434.93	-----
**II**	423.12	433.92	-----
**III**	422.97	433.85	-----
**IV**	422.86	433.65	-----
**V**	421.74	432.47	-----
Hexaazidocyclotriphosphazene	444.43	451.71	455.16

**Table 9.** Calculated relative total energies and the frontier orbital energies for five isomers of diaminohexaazidocyclotetraphosphazene (kcal·mol^−1^) at the B3LYP/6-31G** level of theory ###.

**Compounds**	**E_total_**	**E_LUMO_**	**E_HOMO_**	**ΔE_L-H_**
**I**	2.12	−1.56	−6.01	4.46
**II**	0.00	−1.46	−6.18	4.72
**III**	1.01	−1.50	−6.19	4.69
**IV**	1.15	−1.47	−6.06	4.59
**V**	1.29	−1.54	−6.12	4.58

### In the table, E_total_ means total energy. E_LUMO_ and E_HOMO_ is the energy of the HOMO and LUMO, respectively. ΔE_L-H_ means the gap of E_LUMO_ and E_HOMO_.
